# Deep Learning-Based 3D Reconstruction for Defect Detection in Shipbuilding Sub-Assemblies

**DOI:** 10.3390/s26020660

**Published:** 2026-01-19

**Authors:** Paula Arcano-Bea, Agustín García-Fischer, Pedro-Pablo Gómez-González, Francisco Zayas-Gato, José Luis Calvo-Rolle, Héctor Quintián

**Affiliations:** Department of Industrial Engineering, University of A Coruña, CTC, CITIC, 15403 Ferrol, Spain; paula.arcano@udc.es (P.A.-B.); agustin.garciaf@udc.es (A.G.-F.); pedro.pablo.gomez@udc.es (P.-P.G.-G.); jlcalvo@udc.es (J.L.C.-R.); hector.quintian@udc.es (H.Q.)

**Keywords:** 3D point clouds, overshooting defects, unsupervised anomaly detection, reconstruction-based autoencoders, Isolation Forest, shipbuilding, quality control, unsupervised learning

## Abstract

Overshooting defects in shipbuilding subassemblies are essential to ensure the final product’s overall integrity and safety. In this work, we focus on the automatic detection of overshooting defects in simple and T-shaped sub-assemblies by employing reconstruction-based unsupervised learning on 3D point clouds. To this purpose, we implemented and compared four state-of-the-art architectures, including a Variational Autoencoder (VAE), FoldingNet, a Dynamic Graph CNN (DGCNN) autoencoder, and a PointNet++ autoencoder. These architectures were trained exclusively on defect-free samples, anticipating the possibility of overshooting defects occurring in different locations and with varying geometric patterns that are difficult to characterize explicitly in advance. Those defects are then identified by applying an Isolation Forest to the reconstruction error features, enabling fully unsupervised anomaly detection and allowing us to study how the detection performance changes with the contamination parameter. The results show that reconstruction-based anomaly detection on point clouds is a viable strategy for identifying defects in an industrial environment and the importance of choosing architectures that balance detection performance, stability across different geometries, and computational cost.

## 1. Introduction

Since the late 19th century, the manufacturing industry has experienced multiple transformations. The shift from manual techniques to automated systems was driven by mechanization and steam power [1].

Each subsequent industrial revolution has brought groundbreaking technologies that have progressively reshaped how goods are produced, distributed, and consumed. From electricity and assembly lines in the early 20th century to computer-controlled automation in the latter half. Today, the integration of digital technologies into manufacturing processes has reached an unprecedented scale, fundamentally altering operational efficiency, resource management, and environmental performance [2].

Recent evidence suggests that the digitalization of manufacturing has significantly improved the carbon emission efficiency of exports, demonstrating that modern technological integration not only enhances productivity but also contributes to more sustainable industrial practices [3].

Moreover, the presence of digital industries in urban areas has been shown to promote innovation ecosystems that speed up the development and adoption of new technologies [2].

This shift toward digitally enabled manufacturing is also reshaping global competitiveness, as nations and regions that successfully combine innovative capacity with advanced digitalization levels are better positioned to lead in international markets [4]. In this context, understanding how to employ emerging technologies to optimize manufacturing processes and overcome quality control challenges has become a major concern for industry and academia as well.

These technological advances have become essential in the shipbuilding sector, where they have boosted the quality of final products and streamlined production workflows. Modern defect detection technologies now enable shipyards to perform precise inspections at various stages of assembly, which makes it possible to detect deviations early on and reduce the need for costly rework [5].

Quality control processes have thus evolved from traditional manual inspections to highly integrated systems that monitor dimensional accuracy, structural integrity, and compliance with design specifications throughout the entire construction cycle. Implementing these frameworks reduces defects, improves customer satisfaction, and optimizes resource allocation by identifying inefficiencies before they propagate throughout the process [6].

These digital technologies are being adopted globally at an accelerating rate, with shipyards in many regions reporting significant improvements in traceability, production efficiency, and defect management. For example, smart factory initiatives in China have shown that using intelligent initial design models combined with data-driven case analysis can substantially reduce production time and improve coordination between departments [7].

However, the competitive environment remains challenging, especially for European shipyards. They face pressure from lower-cost producers in Asia while trying to uphold their reputation for building technologically sophisticated, high-quality vessels [8].

To remain competitive, many European shipyards have adopted a collaborative model that relies on an extensive network of subcontractors to supply specialized components and perform specific manufacturing tasks. This approach enables shipyards to focus on system integration and final assembly while harnessing the expertise and flexibility of smaller suppliers. It also distributes financial risk and allows for quicker adaptation to market fluctuations and changing regulatory standards [9].

Given the massive scale and complexity of modern vessels, the early detection of defects in smaller structural components is essential to guaranteeing the final product’s overall integrity and safety. A typical ship is assembled hierarchically from thousands of individual elements, beginning with one of the smallest fabricated units known as simple sub-assemblies and progressing to more complex structures called T sub-assemblies. These fundamental components are then grouped and welded together to create larger modular blocks; these blocks are assembled into the main hull sections and other primary divisions of the vessel.

Because this construction process relies on the precise fit and alignment of countless prefabricated pieces, even minor dimensional deviations or weld defects at the subassembly level can propagate upward through the assembly hierarchy, leading to misalignment, structural weakness, or the need for extensive and costly rework during block integration.

Defects that remain undetected in the early stages of production may compromise the ship’s structural performance under operational loads, which could affect its long-term durability, reliability, and fuel efficiency. Therefore, it is essential to implement strict quality control measures during sub-assembly and block fabrication stages. This ensures that production schedules are maintained, costs are controlled, and the final vessel meets strict safety standards and regulatory requirements.

Machine learning and computer vision techniques have emerged as powerful tools for automating defect detection across various industrial domains [10]. These techniques offer substantial improvements in inspection speed, consistency, and accuracy compared to manual methods. These data-driven approaches can identify patterns and anomalies that human inspectors might overlook, especially when working with large volumes of components or complex geometric structures [11].

Within the broader field of automated inspection, point cloud processing has gained significant attention as a powerful method for capturing and analyzing three-dimensional geometric information with high precision. Generated through laser scanning or photogrammetry, point clouds provide dense spatial representations of physical objects, enabling detailed analysis of surface geometry, dimensional conformance, and structural integrity [12]. Applying machine learning algorithms directly to point cloud representations makes it possible to detect deviations from nominal geometry, identify surface defects, and assess assembly quality entirely automatically.

One particularly promising approach within this domain involves the use of reconstruction-based models, where a neural network learns to encode the normal geometric characteristics of defect-free components and then attempts to reconstruct input point clouds. In this paradigm, regions of the input that deviate significantly from the learned distribution, such as dents, warping, or misaligned features, result in higher reconstruction errors, which can be interpreted as indicators of potential defects [13].

Recent advancements in deep learning have demonstrated that reconstruction-based architectures are highly effective for unsupervised defect detection, where models are trained exclusively on defect-free samples without requiring labeled examples of anomalies [14]. These architectures learn compact latent representations that capture the underlying distribution of normal data. During inference, they identify inputs that significantly deviate from this learned distribution by resulting in elevated reconstruction errors.

The value of this unsupervised strategy is especially high in manufacturing environments where there is a scarcity and high cost of labeled defect data, as the model can be trained only on examples of acceptable components and subsequently used for the identification of anomalies during production. While several studies have addressed defect detection in shipbuilding, most existing approaches depend on traditional inspection protocols or human expertise. There has been limited exploration of using advanced deep learning architectures with three-dimensional geometric data. For example, research on traditional shipbuilding practices has used logic-based analysis frameworks to systematically identify potential failure modes [15]. Similarly, deep learning has been applied to detect painting defects in shipyards using image-based convolutional networks [16], and machine learning classifiers have been deployed for automated weld inspection based on sensor data [17], yet none of these studies have researched the application of point cloud autoencoders or reconstruction-based anomaly detection models for shipbuilding components.

The use of 3D reconstruction and point cloud-based methods for defect detection has become popular in many manufacturing sectors. For example, in additive manufacturing, there is a case in which they employ in situ point cloud processing combined with machine learning to rapidly identify surface defects during printing, enabling real-time quality monitoring and early intervention [18].

Similarly, depth-based approaches have been developed to enhance anomaly detection in industrial components by simulating depth information to augment training data and improve model robustness [19]. Additionally, lightweight network architectures specifically designed for 3D industrial anomaly detection have demonstrated that efficient point cloud processing can achieve high accuracy while maintaining computational feasibility for deployment in production environments [20].

Recent studies have shown that deep learning-based 3D reconstruction can be effectively employed for defect detection in industrial components. For example, a recent 3D anomaly detection method based solely on point cloud reconstruction demonstrates that autoencoder-style reconstruction can localize industrial surface defects without relying on large memory banks or pre-trained models [21]. Other authors propose full point-cloud reconstruction pipelines for 3D anomaly detection on industrial parts, demonstrating competitive performance on datasets such as MVTec 3D-AD [22]. Reconstruction-based autoencoders have also been applied to surface inspection scenarios, where deviations are detected by comparing reconstructed and measured shapes from laser sensors [23]. In parallel, several reviews highlight the growing role of point-cloud deep learning in industrial production and defect inspection, with reconstruction-based methods emerging as a key direction for automatic quality control [24,25].

Unlike these works, which mainly focus on generic benchmark datasets or other manufacturing domains, the application of reconstruction-based point cloud models to real industrial components presents an opportunity to extend their use to more complex and critical scenarios, such as shipbuilding sub-assemblies.

This work focuses on the use of point cloud reconstruction algorithms to detect overshooting defects in 3D printed representations of simple and T subassemblies, whose blueprints were provided by a company working as a subcontractor to a shipyard. To achieve this, we will evaluate a few different unsupervised learning architectures, each trained to model the geometry of defect-free samples. Following reconstruction, an Isolation Forest algorithm is applied to the resulting error metrics to classify defective components. The purpose of this study is to compare several approaches and identify suitable options for automated overshooting detection in a realistic industrial setting. Our goal is to offer practical insights for implementing reliable quality control in shipbuilding and other high-precision manufacturing environments.

Based on this context, the main contributions of this work are threefold. First, we propose an unsupervised, reconstruction-based pipeline for detecting overshooting defects in shipbuilding sub-assemblies using 3D point clouds and anomaly scores derived from reconstruction errors. Second, we systematically compare several deep learning architectures for point cloud reconstruction under a common training and evaluation setup. We analyze their convergence behavior, computational cost, and detection performance. Third, we demonstrate the practical relevance of this approach on multiple sub-assembly geometries, showing how the choice of architecture and anomaly threshold affects the trade-off between defect detection capability and false alarms, and providing guidelines for its adoption in industrial quality control.

This paper is structured in six main sections. After the introduction, Section 2 describes the industrial context and characteristics of the case study components. In Section 3 we present our approach. Section 4 describes the methods and materials used. Section 5 presents the experimental setup, implementation details, results, and comparative analysis. Section 6 concludes with a discussion and recommendations for future research directions.

## 2. Case Study

A ship’s structure is built according to a hierarchical assembly process. The vessel is divided into large blocks, each of which is composed of smaller sub-blocks. These sub-blocks are assembled from multiple types of subassemblies, which are manufactured separately and joined through welding and fitting operations to form the final structural units. Among these elements, the T-subassemblies represent critical structural components located at the junctions where transverse and longitudinal beams intersect. They play an essential role in ensuring the vessel’s overall stiffness and load-bearing capacity, and therefore require high precision in their fabrication.

For this study, a company that works as an auxiliary supplier for a shipyard provided us with the official blueprints of a few representative T-subassemblies, as the original components are too large to handle directly. These parts were selected due to their complex geometry and the frequent occurrence of dimensional deviations during manufacturing. One of the most common issues observed during production is the overshooting defect, which occurs when material is unintentionally removed beyond the intended design surface during the cutting process. This typically happens when the thermal or mechanical cutting tool slightly exceeds the programmed trajectory, or when positioning or alignment errors cause the torch or blade to advance too far. As a result, small recesses or notches are generated along the edge, where the actual contour lies inside the nominal boundary, reducing the effective contact area at welded joints and potentially affecting both assembly precision and structural integrity.

These deviations can alter the geometry of the part, which can affect the precision of the assembly and the overall structural integrity of the vessel.

The identification of such defects is essential, as even minor deviations have the potential to compromise the assembly process and the structural performance of the parts. To address this challenge, we propose a system that leverages 3D point cloud data to identify anomalies in the geometry of the T-subassemblies.

The approach proposed is unsupervised, thereby eliminating the necessity for labeled defective samples during the training phase. This is of particular importance in industrial settings, where defects are often highly variable and unpredictable, making it impractical to collect representative datasets of all possible failure scenarios.

**Figure 1:**
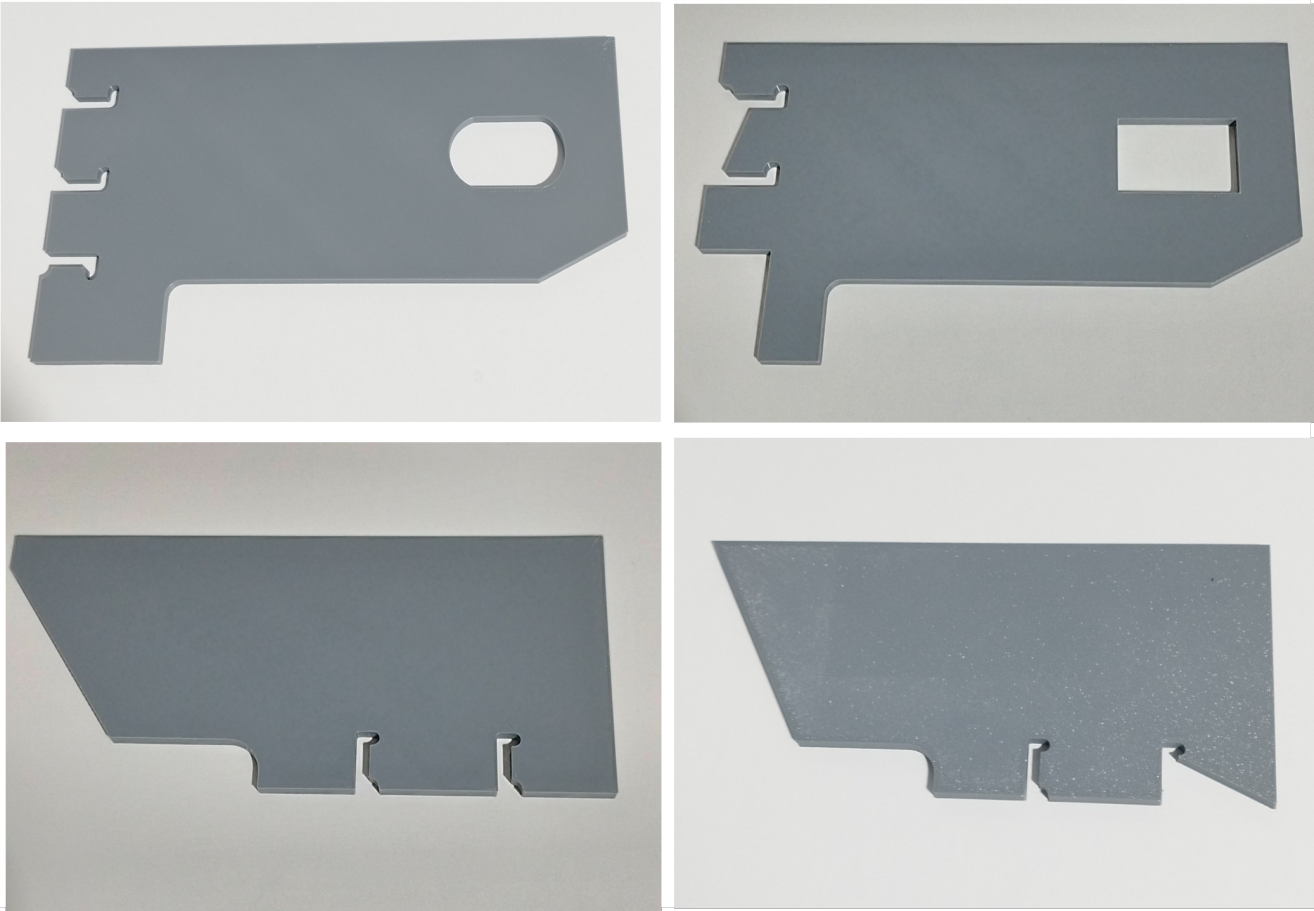
Pictures of non-defective (**right**) and defective (**left**) 3D-printed pieces.

To this end, the T-subassemblies in question were reproduced through 3D printing, a process that was based on the original blueprints provided by the company. The models were fabricated using a fused deposition modeling (FDM) printer and polylactic acid (PLA) filament, which were selected for their ease of use, dimensional stability, and cost efficiency. This process enabled the fabrication of precise replicas that maintained the original designs’ overall geometry while exhibiting substantial dimensional reduction and enhanced manageability. Figure 1 shows a few pictures of these replicas. The pieces on the right resemble the non-defective ones, while the pieces on the left include some overshooting defects.

## 3. Approach

**Figure 2:**
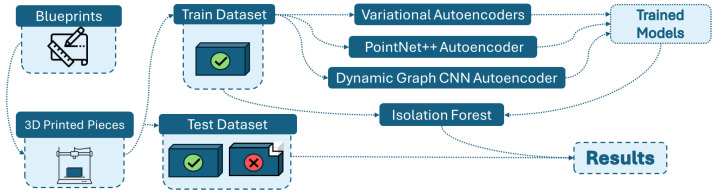
Visual representation of the approach.

Our approach follows a four-stage reconstruction-based anomaly detection pipeline, illustrated in Figure 2. The main stages are: (1) component modeling and defect definition, (2) point cloud acquisition and dataset construction, (3) training of point cloud reconstruction models on non-defective data, and (4) anomaly detection using Isolation Forest on reconstruction error features.

For our approach, we relied on the company’s official blueprints of three different components, referred to as T-subassemblies. These parts are characterized by their large size and considerable weight, with lengths ranging from 3 to 5 m and masses between 181 and 931 kg. Due to their dimensions, the handling or manipulation of the original pieces was impractical. Therefore, we used 3D printing technology to reproduce the components, which allowed for quicker prototyping while maintaining the geometric fidelity of the original designs.

To make the replicas easier to manipulate and to fit within the limitations of the 3D printing equipment, we uniformly scaled the models down to 5% of their original dimensions, reducing, for example, a 4 m component to approximately 20 cm in length. Additionally, based on the blueprint geometry, we generated versions of the components that intentionally incorporated overshooting defects. These defects were designed to look like the imperfections seen in real manufacturing processes, so we could analyze and compare the effect of these defects in a controlled experimental setting.

Once the 3D printed pieces were ready, our next step was to capture their geometric information as point clouds. To do this, we used an Intel RealSense D455 depth camera to get multiple point clouds for each component from different positions and orientations. This process was done for both non-defective and defective versions of the pieces. We collected data from different viewpoints and placements. This allowed us to better replicate realistic acquisition conditions, with the aim of improving the model’s capacity to generalize and adapt to diverse real-world scenarios. After the data was collected, the point clouds corresponding to the non-defective components were randomly divided into three subsets: 70% for training, 20% for testing, and 10% for validation. In contrast, all defective component samples were used exclusively during the validation phase. This data partitioning scheme was designed according to the unsupervised nature of our approach to ensure that the model was trained solely on non-defective data and that its ability to identify defects was evaluated independently.

In the third stage (model training), we selected four different architectures commonly used for unsupervised learning on 3D point cloud data reconstruction such as a Variational Autoencoder (VAE), FoldingNet, Dynamic Graph CNN (DGCNN) Autoencoder and PointNet++ Autoencoder. These models were chosen to provide a diverse representation of state-of-the-art approaches in point cloud feature extraction and reconstruction. By comparing their performance under the same experimental conditions, we aimed to evaluate how different geometric encoding strategies influence the model’s ability to represent complex shapes and to distinguish between defective and non-defective components.

In the final stage (anomaly detection), after training the models, we computed reconstruction metrics for each sample, specifically the Earth Mover’s Distance (EMD) and the Chamfer Distance (CD), to quantify the similarity between the input and reconstructed point clouds. We then used these metrics as input features for the Isolation Forest algorithm to perform anomaly detection. The Isolation Forest was trained exclusively on the reconstruction metrics obtained from the non-defective components, allowing it to learn the distribution of normal reconstruction errors.

## 4. Methods and Materials

The following section will explain all the methods that were used in this research study.

### 4.1. Point Cloud Acquisition

For the acquisition of the 3D point-cloud data, we used an Intel RealSense D455 Depth Camera.(Intel, Santa Clara, USA) This device employs a stereo-vision depth sensing architecture, combining a left and a right imager, together with an infrared projector that emits a non-visible static IR pattern to enhance depth accuracy in low-texture or challenging surfaces.

The D455 is capable of capturing depth maps at resolutions up to 1280 × 720 pixels and frame rates of up to 90 frames per second, with an approximate field of view of 86° × 57° (horizontally and vertically). Its effective operational range extends from about 0.4 m to 6 m, depending on lighting and surface reflectivity. The camera also includes an RGB sensor and an inertial measurement unit (IMU), which allows for color alignment and pose tracking during acquisition [26].

In a stereo vision system, such as the one used by the D455, depth perception is achieved by analyzing small differences between two images captured simultaneously from slightly different viewpoints. These differences, known as disparities, directly relate to the distance of each point in the scene, meaning the greater the disparity, the closer the object is to the cameras. However, matching corresponding points between the two images becomes difficult in regions where the surface lacks sufficient visual texture or distinct features, reducing depth accuracy.

To mitigate this limitation, the D455’s integrated infrared (IR) projector emits a structured, invisible light pattern onto the observed surface. This artificial pattern introduces additional texture, enhancing the stereo matching algorithm’s ability to find reliable correspondences between the left and right images, even under uniform or low-contrast lighting conditions. Combining passive stereo imaging with active infrared projection gives the D455 the ability to achieve steady and reliable depth estimation across a wide variety of materials and lighting conditions.

**Figure 3:**
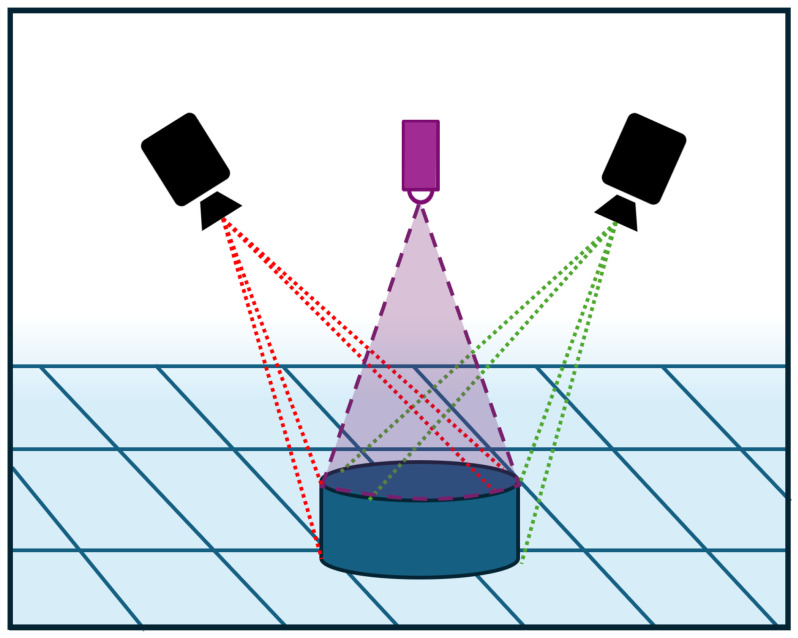
Visual representation of the operating principle of the Intel RealSense D455 Camera.

Figure 3 illustrates the operating principle of the Intel RealSense D455 camera.

### 4.2. Models

In our study, we selected autoencoder-based models for 3D reconstruction and anomaly detection. Unlike feature-based methods, which detect anomalies at the level of extracted descriptors, reconstruction-based approaches operate directly on the point-level geometry of the data. Autoencoders are trained only on anomaly-free samples; they use an encoder to compress the input point cloud into a latent representation and a decoder to reconstruct it. During inference, the model attempts to reconstruct both normal and defective surfaces; however, since it only saw normal data during training, it struggles to accurately reproduce unseen anomalous regions. The discrepancies between the original and reconstructed point clouds are then used to calculate anomaly scores, identifying deviations from the expected geometry [14].

In the following subsections, we describe each reconstruction model using a local mathematical notation. All symbols such as *X*, $\hat{X}$, *z*, *U*, and *N* are defined within the context of each model and their meaning does not extend beyond the corresponding subsection.

#### 4.2.1. Variational Autoencoder (VAE)

Variational Autoencoders (VAEs) are generative models designed to learn a compact, continuous representation of complex data distributions.

When applied to 3D point clouds [13], a VAE first transforms the raw input points into a latent space through an encoder network. The encoder processes each point individually using successive layers that capture local and global geometric features, followed by an aggregation step, typically a global pooling operation, to extract information from individual points and their neighborhoods, allowing the model to encode both fine details and overall shape structure. This global feature vector is then mapped to two vectors representing the mean and log-variance of the latent Gaussian distribution.

A key feature of the VAE is the reparameterization trick, which allows a latent vector to be sampled from the Gaussian distribution in a differentiable way, preserving the flow of gradients for optimization. This latent vector serves as a compact representation of the original point cloud, encoding the essential geometric features learned from normal, anomaly-free samples.

Formally, the encoder is defined as

(1) $$q_{\phi }\left(z|X\right)=N\left(z;\;\mu \left(X\right),\mathrm{diag}(\sigma^{2}\left(X\right))\right),$$

with

(2) z=μ(X)+σ(X)⊙ϵ,ϵ∼N(0,I),

where:

- $X\in R^{N\times 3}$ is the input 3D point cloud.
- *N* is the number of points in the point cloud.
- $z\in R^{d}$ is the latent vector.
- *d* is the dimensionality of the latent space.
- $\mu \left(X\right)\in R^{d}$ is the mean of the approximate posterior Gaussian distribution.
- $\sigma^{2}\left(X\right)\in R^{d}$ is the diagonal variance of the approximate posterior.
- ϵ∼N(0,I) is standard Gaussian noise.
- ϕ denotes the parameters of the encoder network (weights and biases).

The decoder then takes this latent vector and reconstructs the point cloud in a stepwise manner. It gradually transforms the compressed information into the 3D coordinates of the output points, reassembling the global structure while attempting to preserve local details.

Formally, the decoder is defined as

(3) $$\hat{X}=g_{\theta }\left(z\right),$$

where:

- $z\in R^{d}$ is the latent vector sampled from the encoder.
- $\hat{X}\in R^{N\times 3}$ is the reconstructed 3D point cloud.
- *N* is the number of points in the output point cloud.
- *d* is the dimensionality of the latent space.
- $g_{\theta }\left(\cdot \right)$ denotes the decoder neural network with parameters θ (weights and biases).

Through this encoding–decoding architecture, VAEs are capable of representing complex 3D geometries in a continuous latent space, allowing for the reconstruction of familiar patterns while inherently struggling to reproduce regions that deviate from the learned distribution.

#### 4.2.2. FoldingNet (FN)

FoldingNet is an autoencoder created to learn compact representations of three-dimensional point clouds through a combination of local feature extraction and a decoding process that incorporates geometric reasoning [27]. The encoder follows the idea of PointNet [28], where each point is processed independently with shared multilayer perceptrons. These pointwise features are then gathered through a global max pooling step, which produces a single latent vector that summarizes the overall structure of the cloud without requiring information about how the points are connected.

Formally, the encoder is defined as

(4) $$z=f_{\phi }\left(X\right)=\gamma \;\left(\;\overset{N}{\underset{i=1}{\mathrm{pool}}}\psi \left(x_{i}\right)\right),$$

where:

- $X={\left\{x_{i}\right\}}_{i=1}^{N}$, with $x_{i}\in R^{3}$, is the input 3D point cloud;
- ψ(·) is a point-wise multilayer perceptron applied independently to each point $x_{i}$;
- pool is a symmetric aggregation function (e.g., max-pooling) over all points;
- γ(·) is a final multilayer perceptron that maps the aggregated feature to the latent space;
- $z\in R^{d}$ is the resulting global latent feature vector;
- ϕ denotes all learnable parameters of the encoder network.

The name FoldingNet comes from the strategy used in the decoder. Instead of predicting the three-dimensional coordinates directly, the network begins with a simple two-dimensional grid and learns how to fold this grid into the shape of the target object. The decoder performs this transformation in two steps, each implemented with fully connected layers that gradually bend and reshape the planar grid so that it approaches the matches of the input cloud. Through this process, the model can recover the overall surface of the object and produce a reconstruction that follows its main structural features.

Formally, the decoder is defined as

(5) $$\hat{X}=G_{\theta }\left(U,z\right)={\left\{{\hat{x}}_{i}\right\}}_{i=1}^{M},\;{\hat{x}}_{i}=g_{\theta }\left(u_{i},z\right),\;i=1,\ldots ,M,$$

where:

- $U={\left\{u_{i}\right\}}_{i=1}^{M}$, with $u_{i}\in R^{2}$, is a fixed 2D canonical grid of *M* points;
- $z\in R^{d}$ is the latent feature vector produced by the encoder;
- $g_{\theta }\left(\cdot ,\cdot \right)$ is the point-wise folding network (an MLP that maps $\left(u_{i},z\right)$ to a 3D point) parameterized by θ;
- $G_{\theta }\left(U,z\right)$ denotes the decoder function that applies $g_{\theta }$ to all grid points in *U* to produce the full reconstruction;
- ${\hat{x}}_{i}\in R^{3}$ is the reconstructed 3D point corresponding to grid point $u_{i}$;
- $\hat{X}={\left\{{\hat{x}}_{i}\right\}}_{i=1}^{M}\in R^{M\times 3}$ is the reconstructed 3D point cloud.

#### 4.2.3. Dynamic Graph CNN (DGCNN)

The Dynamic Graph Convolutional Neural Network introduces a way to process point clouds by updating the neighborhood structure of the data throughout the network [29]. Its encoder is built around the idea of computing edge features, where each point is compared with its nearest neighbors in feature space rather than in the original coordinate space. This dynamic construction of local graphs is repeated at every layer, allowing the network to capture relationships that change as the representation becomes more abstract. By aggregating these edge features and progressively increasing the receptive field, the encoder produces a latent vector that reflects both the global shape of the object and the local geometric patterns present in the cloud. Formally, the encoder is defined as

(6) $$h_{i}^{\left(l+1\right)}=\max_{j\in N(i)}\;\phi^{\left(l\right)}\;\left(h_{i}^{\left(l\right)},\;h_{j}^{\left(l\right)}-h_{i}^{\left(l\right)}\right),$$

with

(7) $$z=\max_{i=1}^{N}h_{i}^{\left(L\right)},$$

where:

- $X={\left\{x_{i}\right\}}_{i=1}^{N}$, $x_{i}\in R^{3}$, is the input 3D point cloud;
- $h_{i}^{\left(0\right)}=x_{i}$ and $h_{i}^{\left(l\right)}$ is the feature of point *i* at layer *l*;
- N(i) is the set of *k*-nearest neighbors of point *i* in feature space;
- $\phi^{\left(l\right)}\left(\cdot ,\cdot \right)$ is an MLP at layer *l* (the EdgeConv operator);
- *L* is the number of EdgeConv layers;
- $z\in R^{d}$ is the global latent feature vector.

To reconstruct the point cloud, the decoder takes this latent representation and maps it back to three-dimensional coordinates using a sequence of fully connected layers. Although this decoding stage is simpler than the encoder, it is sufficient to recover the main structure of the shape because the latent space already encodes the key geometric cues extracted through the dynamic graph updates. In this way, DGCNN can be used as an autoencoder, where the dynamic treatment of neighborhoods in the encoder allows the model to adapt to variations in local geometry and produce reconstructions that follow the underlying shape of the input cloud. Formally, the decoder is defined as

(8) $$\hat{X}=g_{\theta }\left(z\right),$$

where:

- $z\in R^{d}$ is the latent vector produced by the encoder;
- $g_{\theta }\left(\cdot \right)$ is a multilayer perceptron parameterized by θ;
- $\hat{X}\in R^{N\times 3}$ is the reconstructed 3D point cloud.

#### 4.2.4. PointNet++ Autoencoder (PN++AE)

PointNet++ [30] is based on the original PointNet [28] architecture, introducing a hierarchical strategy that learns features directly from point sets. This strategy explicitly constructs local neighborhoods in metric space and progressively integrates them into higher-level representations. In the autoencoder configuration, the encoder follows the standard PointNet++ design. The input point cloud is repeatedly subsampled through set abstraction layers. At each stage, local regions are formed around sampled points using distance-based queries. Within each region, shared multilayer perceptrons and a symmetric aggregation function are applied to produce features that describe the local geometry. As these layers are stacked, the network captures larger and larger receptive fields. Finally, the deepest representation combines information about detailed features and the overall object structure. A final global pooling operation compresses these features into a latent vector that serves as a compact descriptor of the entire point cloud.

PointNet++ can be transformed into an autoencoder [31] by combining it with a decoder that maps the latent vector back to three-dimensional coordinates. The encoder component follows the standard PointNet++ design, in which set abstraction layers progressively downsample and aggregate the input point cloud into a compact latent representation. This latent vector acts as a learned bottleneck, forcing the network to compress essential geometric information about the input shape into a fixed-size vector. This process effectively learns a continuous representation of the point cloud distribution. Formally, the encoder is defined as

(9) $$z=\gamma \left(\max_{j=1,\ldots ,M_{L}}f_{j}^{\left(L\right)}\right),$$

where each Set Abstraction (SA) layer operates as

(10) $${\left\{c_{j}^{\left(l\right)},f_{j}^{\left(l\right)}\right\}}_{j=1}^{M_{l}}={\mathrm{SA}}^{\left(l\right)}\left({\left\{x_{i}^{\left(l-1\right)},f_{i}^{\left(l-1\right)}\right\}}_{i=1}^{N_{l-1}}\right),$$

with

(11) $$c_{j}^{\left(l\right)}=\mathrm{FPS}\left(\{x_{i}^{\left(l-1\right)}\}\right),\;N_{j}^{\left(l\right)}=\mathrm{KNN}\left(c_{j}^{\left(l\right)},k\right),\;f_{j}^{\left(l\right)}=\max_{i\in N_{j}^{\left(l\right)}}\psi^{\left(l\right)}\left(x_{i}^{\left(l-1\right)}-c_{j}^{\left(l\right)},f_{i}^{\left(l-1\right)}\right),$$

where:

- $X={\left\{x_{i}^{\left(0\right)}\right\}}_{i=1}^{N}$, $x_{i}^{\left(0\right)}\in R^{3}$, is the input 3D point cloud;
- $f_{i}^{\left(0\right)}=x_{i}^{\left(0\right)}$ are the initial point features;
- FPS(·) denotes farthest point sampling, selecting $M_{l}$ centroids $c_{j}^{\left(l\right)}$;
- $N_{j}^{\left(l\right)}$ is the set of *k* nearest neighbors of centroid $c_{j}^{\left(l\right)}$;
- $\psi^{\left(l\right)}\left(\cdot \right)$ is a point-wise multilayer perceptron at layer *l* (local PointNet);
- $f_{j}^{\left(l\right)}$ is the aggregated local feature for centroid $c_{j}^{\left(l\right)}$;
- *L* is the number of SA layers, with $M_{L}=1$ in the final global layer;
- γ(·) is a fully connected network mapping the global feature to the latent space;
- $z\in R^{d}$ is the final latent vector (bottleneck representation).

The decoder then takes this compressed representation and reverses the encoding process in a simplified form. Rather than mirroring the hierarchical structure of the encoder, the decoder is usually a sequence of fully connected layers that gradually increase the dimensionality of the latent code until it reaches the size needed to represent a fixed number of output points. After the last fully connected layer, the activations are reshaped into a set of three-dimensional coordinates, directly reconstructing the point cloud in Euclidean space. Though the decoding stage is structurally simpler than the encoder and does not explicitly model local neighborhoods or perform set operations, it can recover the main shape and many geometric details because the PointNet++ encoder’s latent space has already organized the data according to multi-scale local patterns and global structure.

Formally, the decoder is defined as

(12) $$\hat{X}=\mathrm{reshape}\left(\phi_{\theta }\left(z\right)\right),$$

where:

- $z\in R^{d}$ is the latent vector produced by the encoder;
- $\phi_{\theta }:R^{d}\to R^{3N}$ is a multilayer perceptron parameterized by θ;
- reshape(·) reshapes the $R^{3N}$ output of $\phi_{\theta }$ into $R^{N\times 3}$;
- $\hat{X}\in R^{N\times 3}$ is the reconstructed 3D point cloud.

#### 4.2.5. Isolation Forest

The Isolation Forest algorithm employs an ensemble of randomly constructed binary trees to model anomalies. These trees are explicitly designed to isolate individual samples rather than model decision boundaries between classes [32]. Each isolation tree is built from a random subsample of the data by recursively partitioning the feature space. At every internal node, an attribute is randomly selected, and a split value is drawn uniformly between the minimum and maximum of that attribute in the current subset. This process continues until the node contains a single instance or the maximum depth is reached. Because anomalous points tend to be rare and lie in low-density regions, they are typically separated from the rest of the data after a few random splits. In this way, normal points, which are embedded in dense clusters, require deeper paths to become isolated. For a given observation, its path length h(x) is defined as the number of edges traversed from the root to the node where the observation is isolated. Taking the average of this length over all trees in the ensemble gives an expectation, E(h(x)), that reflects how easily the observation can be separated from the bulk of the data. The Isolation Forest algorithm models anomalies using an expected path length, which is then converted into a normalized anomaly score comparable across different sample sizes. Let n be the subsample size used to build each tree. Isolation Forest defines a normalization constant c(n) as the average path length of unsuccessful searches in a binary search tree of size n. This constant can be expressed using harmonic numbers as

(13) $$c\left(n\right)=2H\left(n-1\right)-\frac{2(n-1)}{n},$$

where

(14) $$H\left(m\right)=\sum_{k=1}^{m}\frac{1}{k}$$

denotes the *m*-th harmonic number [32]. The anomaly score for a point *x* is then given by

(15) $$s\left(x,n\right)=2^{-\frac{E[h(x)]}{c(n)}},$$

where E[h(x)] is the average path length of *x* over an ensemble of isolation trees. In this formulation, points that are isolated very quickly have small E[h(x)], leading to scores s(x,n) close to 1, while normal points tend to have scores closer to 0. The Isolation Forest algorithm includes a contamination parameter that specifies the expected proportion of anomalies in the dataset. While this parameter does not affect how the trees are constructed or how the score is computed, it directly influences the decision threshold used to classify points as normal or anomalous. When a contamination value is provided, the model selects a cutoff for the anomaly scores so that approximately that fraction of instances are labeled as anomalous. As the contamination value increases, the threshold becomes less strict, resulting in more points being marked as anomalies. However, lower contamination values result in a more conservative selection, considering only the most extreme scores as outliers.

### 4.3. Evaluation Metrics

In this section, we will describe the metrics used to evaluate the performance of our models and discuss how they quantify the similarity or discrepancy between the predicted and reference point clouds.

#### 4.3.1. Earth Mover’s Distance (EMD)

The Earth Mover’s Distance is a measure of dissimilarity between two distributions that can be interpreted as the minimum work required to transform one distribution into the other [33]. In the context of point clouds, each point set is viewed as a discrete distribution of unit masses located in Euclidean space, and EMD computes the optimal way to transport mass from the predicted points to the target points. It is widely used for comparing shapes, point clouds, and histograms because it takes the geometry of the data into account. For two sets of points, the calculation finds the most efficient way to redistribute the mass from one set onto the other, minimizing the overall transportation cost, which depends both on the amount of mass moved and how far it travels. The EMD between two sets of points $P={\left\{p_{i}\right\}}_{i=1}^{m}$ and $Q={\left\{q_{j}\right\}}_{j=1}^{n}$ is defined by:

(16) $$\mathrm{EMD}\left(P,Q\right)=\frac{\sum_{i=1}^{m}\sum_{j=1}^{n}f_{ij}d_{ij}}{\sum_{i=1}^{m}\sum_{j=1}^{n}f_{ij}}$$

where

- $f_{ij}$: the optimal flow of mass from point $p_{i}$ to $q_{j}$;
- $d_{ij}$: the ground distance between $p_{i}$ and $q_{j}$;
- the numerator represents the total cost to match *P* into *Q*;
- the denominator is the total flow, ensuring normalization.

The optimal flow matrix $F=[f_{ij}]$ minimizes the total transportation cost under the constraint that the amount moved from each source and received by each target does not exceed their weights.

#### 4.3.2. Chamfer Distance (CD)

The chamfer distance is a measure of dissimilarity between two point sets that quantifies how well each point in one set can be matched to its closest point in the other set [34]. In the context of point clouds, this metric evaluates the quality of a reconstruction by averaging the distances from each point in the predicted cloud to its nearest neighbor in the ground truth and vice versa. Consequently, the Chamfer Distance effectively reflects spatial correspondence between distributions, even when point counts differ, by penalizing unmatched points and promoting overall shape fidelity. Due to its simplicity and computational efficiency, it is a popular choice in geometric deep learning and shape completion tasks.

The Chamfer Distance between two point sets $P={\left\{p_{i}\right\}}_{i=1}^{m}$ and $Q={\left\{q_{j}\right\}}_{j=1}^{n}$ is defined as:

(17) $$\mathrm{CD}\left(P,Q\right)=\frac{1}{\left|P\right|}\sum_{p\in P}\min_{q\in Q}{∥p-q∥}_{2}^{2}+\frac{1}{\left|Q\right|}\sum_{q\in Q}\min_{p\in P}{∥q-p∥}_{2}^{2}$$

where

- ${∥p-q∥}_{2}^{2}$: squared Euclidean distance between points *p* and *q*;
- $\min_{q\in Q}$: for each *p* in *P*, find the closest point in *Q*;
- Each sum is averaged over the size of its respective set;
- The final value is the sum of the two directed averages, making the metric symmetric.

This metric measures the average closest-point distance between two point clouds in both directions, providing a symmetric but non-metric approximation of their geometric similarity.

## 5. Experiments and Results

The following section will include a description of the experiments that were performed and the results of those experiments.

### 5.1. Experiments Setup

To ensure a fair comparison across all architectures, we employed a consistent experimental setup for training and evaluation. All models were trained using 200 epochs and the Chamfer Distance as the reconstruction loss function [35], processing point clouds with 4096 points per sample. Training was performed with a batch size of 8 and a learning rate of $1\times 10^{-4}$, using the Adam optimizer. All experiments were performed on an NVIDIA RTX A6000 GPU, and models were implemented in TensorFlow/Keras. This uniform training configuration allows for direct performance comparison under identical conditions, isolating the impact of architectural differences on defect detection capability.

**Variational Autoencoder (VAE):** Following the architecture described in Section 4, the encoder processes the input point cloud through three 1D convolutional layers with 64, 128, and 256 filters, each followed by batch normalization and ReLU activation. After global max pooling, the aggregated features are projected into two 128-dimensional vectors representing μ(X) and $\sigma^{2}\left(X\right)$ of the latent distribution. The latent vector *z* is sampled using the reparameterization trick as z=μ(X)+σ(X)⊙ϵ with ϵ∼N(0,I), where the log-variance is clipped to [−10,10] to ensure numerical stability. The decoder then expands this latent representation through fully connected layers of dimensions 512 and 1024, both with batch normalization and ReLU activations, before projecting to 4096×3 output coordinates.

**FoldingNet (FN):** As mentioned in Section 3, the encoder follows the PointNet structure with three 1D convolutional layers (64, 128, and 512 filters) followed by global max pooling and a fully connected layer that produces a 128-dimensional latent code. The decoder begins with a fixed 2D grid of size $\sqrt{4096}\times \sqrt{4096}$ with coordinates in [−0.5,0.5]. This grid is tiled across the batch and concatenated with the expanded latent vector at each point. The concatenated representation is then processed through two fully connected layers with 512 neurons and ReLU activation, progressively folding the 2D grid into 3D space. The final layer outputs 3 coordinates per point, reconstructing the target point cloud through this learned deformation process.

**Dynamic Graph CNN (DGCNN):** Consistent with the formulation in Section 3, the encoder constructs dynamic graphs at each layer by computing edge features between points and their *k*-nearest neighbors in feature space. Four edge convolutional layers with 64, 64, 128, and 256 filters are applied, where each EdgeConv operation computes $h_{i}^{\left(l+1\right)}=\max_{j\in N(i)}\phi^{\left(l\right)}\left(h_{i}^{\left(l\right)},h_{j}^{\left(l\right)}-h_{i}^{\left(l\right)}\right)$. After global max pooling over all point features, a fully connected layer produces the 128-dimensional latent vector $z=\max_{i=1}^{N}h_{i}^{\left(L\right)}$. The decoder consists of two fully connected layers with 512 and 1024 neurons, followed by a final projection to 4096×3 coordinates. Although the decoder does not employ graph convolutions, the rich latent representation learned through dynamic neighborhood aggregation in the encoder enables accurate reconstruction.

**PointNet++ Autoencoder (PN++AE):** Following the architecture design mentioned in Section 3, the encoder employs three set abstraction layers. The first layer uses Farthest Point Sampling (FPS) to select 1024 centroids, groups each with k=32 nearest neighbors, and applies 1D convolutions with 64, 64, and 128 filters to compute local features. The second layer downsamples to 256 centroids with the same grouping strategy, applying convolutions with 128, 128, and 256 filters. The third layer performs global aggregation with convolutions of 256, 512, and 512 filters, producing a global feature vector. This global representation is mapped through a fully connected layer with 512 neurons and batch normalization to obtain the 128-dimensional latent code. The decoder mirrors the structure used in previous models, employing fully connected layers with 1024 and 2048 neurons, each followed by batch normalization and ReLU activation, before projecting to the final 4096×3 output. This hierarchical encoding strategy enables the model to capture multi-scale geometric information for accurate reconstruction.

**Isolation Forest:** To classify reconstructed samples as defective or non-defective, we apply the Isolation Forest algorithm to the reconstruction errors produced by each model. For every model and reference part, we first compute the Chamfer Distance and Earth Mover’s Distance between the input and its reconstruction on the training (defect-free) set and the test (defective) set, storing these values as two-dimensional feature vectors. The Isolation Forest is then trained exclusively on the error distributions of normal samples, treating (CD and EMD) as input features in an unsupervised setting.

Once fitted, the model is evaluated on the combined set of normal and defective samples by predicting anomaly labels and computing an anomaly score for each observation. To study the sensitivity of the detector, we sweep the contamination parameter from 0.001 to 0.5 in steps of 0.001, generating a separate model and evaluation for each value. For every contamination level, we report precision, recall, F1-score, and ROC-AUC, together with confusion matrices. This analysis will allow us to quantify the effect of contamination threshold choices on the relationship between correctly identifying overshooting defects and avoiding false alarms across different reconstruction architectures.

### 5.2. Results and Analysis

In this section, we will present the experimental results for all combinations of the reconstruction model and subassembly. As previously mentioned, we considered three different sub-assemblies, which we will refer to as objects 206, 221, and 301 to differentiate them clearly. These identifiers were randomly assigned and do not correspond to any internal naming convention, with the sole purpose of preserving the requested confidentiality of the industrial data.

**Figure 4:**
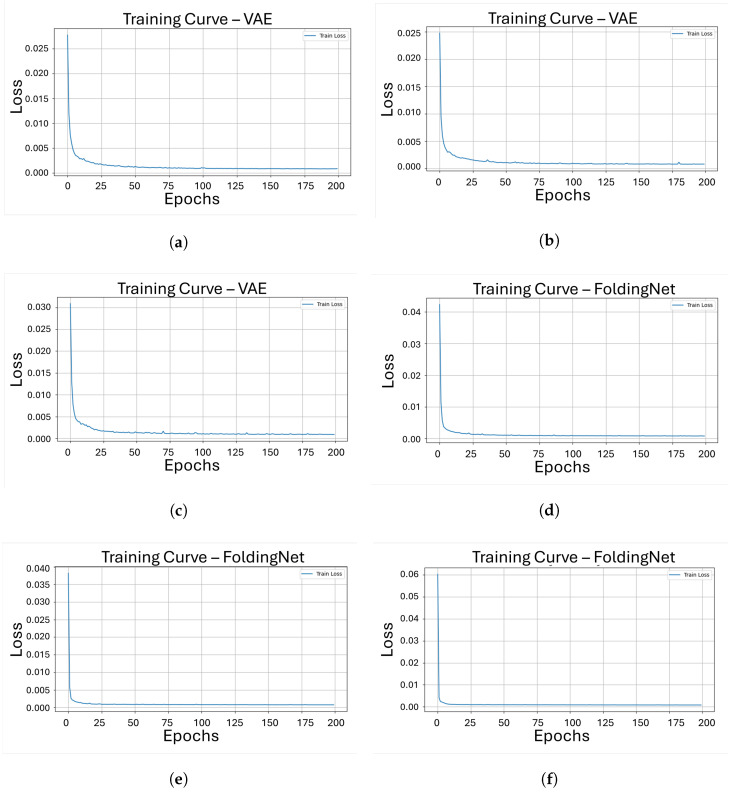

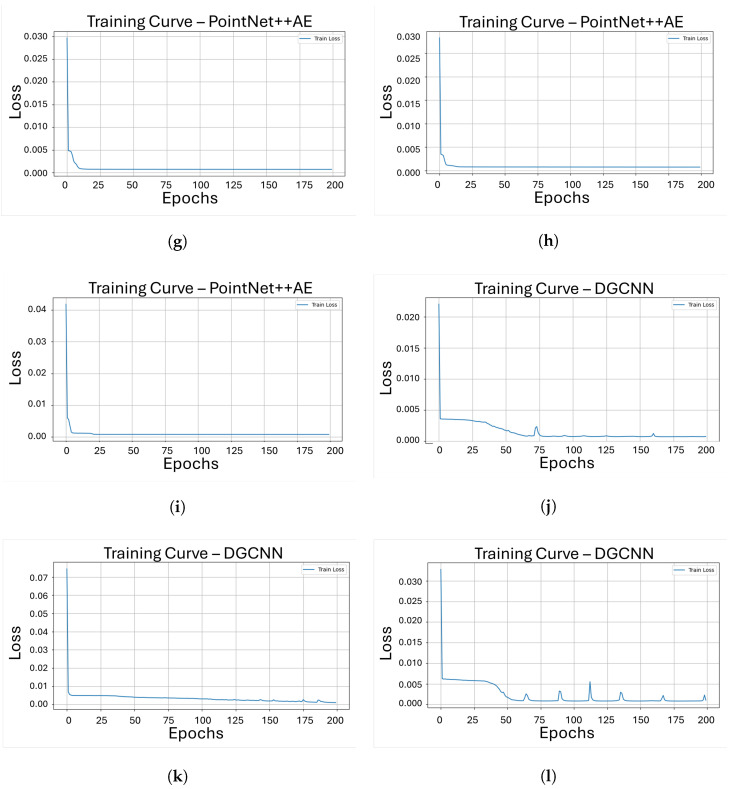
Training curves showing reconstruction loss over 200 epochs for our four architectures on three different pieces. (**a**) Loss vs. epochs for object 206 with VAE; (**b**) Loss vs. epochs for object 221 with VAE; (**c**) Loss vs. epochs for object 301 with VAE; (**d**) Loss vs. epochs for object 206 with FoldingNet; (**e**) Loss vs. epochs for object 221 with FoldingNet; (**f**) Loss vs. epochs for object 301 with FoldingNet; (**g**) Loss vs. epochs for object 206 with PointNet++AE; (**h**) Loss vs. epochs for object 221 with PointNet++AE; (**i**) Loss vs. epochs for object 301 with PointNet++AE; (**j**) Loss vs. epochs for object 206 with DGCNN; (**k**) Loss vs. epochs for object 221 with DGCNN; (**l**) Loss vs. epochs for object 301 with DGCNN.

Figure 4 shows the evolution of the training loss, measured with the Chamfer Distance, over 200 epochs for each model-object pair Figure 4a–c correspond to the VAE model trained on objects 206, 221, and 301, respectively; Figure 4d–f show the same curves for FoldingNet; Figure 4g–i illustrate the behavior of PointNet++ Autoencoder; and Figure 4j–l shows the results for DGCNN. Across all cases, the loss generally decreases as the number of epochs increases, indicating that the models progressively improve their reconstruction capability under the common training setup.

From the loss curves in Figure 4, it can be observed that the VAE, FoldingNet, and PointNet++ Autoencoder exhibit a broadly similar training behavior, with the Chamfer Distance steadily decreasing as the number of epochs increases and converging to low loss values relatively early in the training process. In contrast, the DGCNN shows a less stable trajectory, particularly for objects 206 and 301, where the loss occasionally increases during training, producing noticeable spikes before continuing to decrease. This behavior suggests that the DGCNN model requires more epochs to achieve comparable levels of reconstruction error and that its dynamic graph construction makes the training process more challenging compared to other architectures under the same conditions.

Also, we evaluated the computational characteristics of the four reconstruction models by comparing their training time, number of parameters, and GPU memory usage. Table 1 summarizes these aspects and provides a quantitative view of the relation between model complexity and computational cost.

The average training time represents the mean training duration across the three objects (206, 221, and 301) for each model. Among the evaluated architectures, the VAE is the fastest to train (347.50 s on average), followed by FoldingNet (437.27 s) and DGCNN (554.35 s), while PointNet++AE requires noticeably more time (935.29 s). This tendency is consistent with the relative complexity of the architectures, since PointNet++AE incorporates multiple hierarchical set abstraction layers and a larger number of parameters.

The number of trainable parameters reflects the capacity of each model to learn complex representations from the data. PointNet++AE exhibits the highest parameter count, with 28,403,776 trainable parameters, followed by DGCNN with 14,220,800 and the VAE with 13,426,688 parameters. In contrast, FoldingNet is substantially more compact, with only 602,755 trainable parameters, which explains in part its competitive training time despite operating on point clouds of 4096 points. The non-trainable parameters are negligible in all cases, appearing only in the VAE and PointNet++AE due to the use of batch normalization layers.

Regarding computational load, all models were trained on the same NVIDIA RTX A6000 GPU (Nvidia, Santa Clara, CA, USA) and showed similar VRAM usage, in the range of approximately 45–47 GB as shown in Table 1. PointNet++AE and FoldingNet show slightly higher memory consumption than the VAE and DGCNN, which is consistent with their decoder designs and internal feature representations. Overall, these results highlight that while PointNet++AE offers the highest representational capacity, it does so at the cost of increased training time, whereas FoldingNet provides a more lightweight alternative with lower parameter count and moderate computational demands.

After training all reconstruction models, we proceed to evaluate their effectiveness in the overshooting detection task. To this end, we apply the Isolation Forest algorithm to the reconstruction errors (chamfer distance and earth mover’s distance) of each model/object combination and study how the detection performance changed with the contamination parameter.

**Figure 5:**
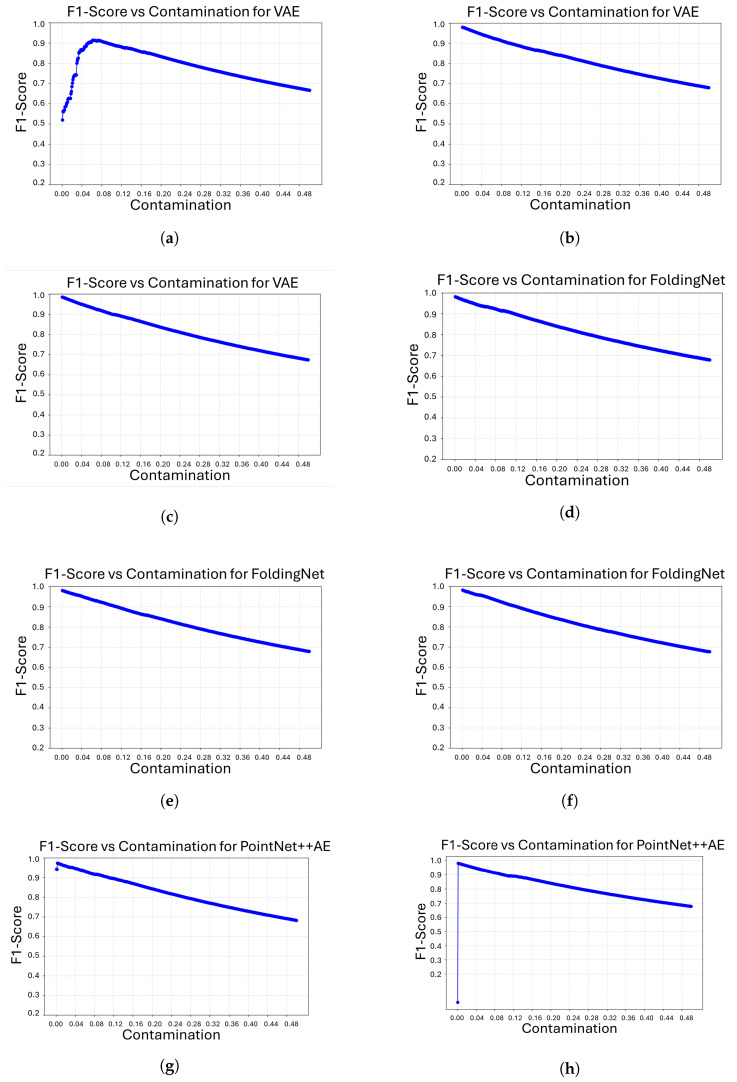

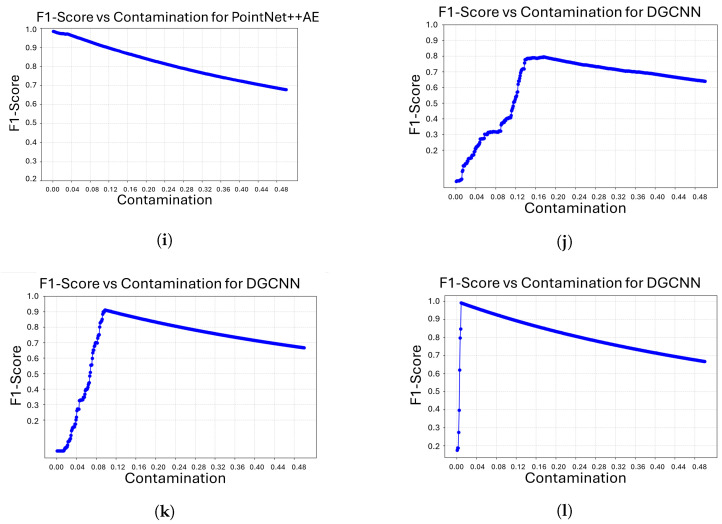
F1-Score vs. Contamination curves for our four architectures on three different objects. (**a**) F1-Score vs. Contamination for object 206 with VAE; (**b**) F1-Score vs. Contamination for object 221 with VAE; (**c**) F1-Score vs. Contamination for object 301 with VAE; (**d**) F1-Score vs. Contamination for object 206 with FoldingNet; (**e**) F1-Score vs. Contamination for object 221 with FoldingNet; (**f**) F1-Score vs. Contamination for object 301 with FoldingNet; (**g**) F1-Score vs. Contamination for object 206 with PointNet++AE; (**h**) F1-Score vs. Contamination for object 221 with PointNet++AE; (**i**) F1-Score vs. Contamination for object 301 with PointNet++AE; (**j**) F1-Score vs. Contamination for object 206 with DGCNN; (**k**) F1-Score vs. Contamination for object 221 with DGCNN; (**l**) F1-Score vs. Contamination for object 301 with DGCNN.

Figure 5 shows the evolution of the F1-score as a function of the contamination parameter for all model/object combinations. In general, all architectures achieve their highest F1 values at relatively low contamination levels, after which performance gradually deteriorates as the assumed proportion of anomalies increases. This behavior is consistent with the role of contamination as a decision threshold in the Isolation Forest algorithm. Overly large contamination values force the classifier to incorrectly label a high number of samples as anomalous, which negatively impacts the balance between precision and recall.

Some differences between models and objects can also be observed. For most cases, the optimal F1-score is reached at very low contamination values, indicating that a conservative anomaly threshold is sufficient to separate defective from non-defective samples. However, the VAE for object 206 requires a slightly higher contamination level to attain its maximum F1-score, suggesting that the reconstruction error distribution for this object is less sharply separated. Similarly, the DGCNN model for objects 221 and, in particular, 206 attains its best F1-scores at higher contamination values than the other architectures, which indicates that its anomaly scores are more dispersed and require a less restrictive threshold to correctly identify overshooting defects.

The best validation performance achieved by each reconstruction model on the three objects is detailed in Table 2. The contamination value that maximizes the F1-score is selected for every model/object pair, and the precision, recall, and ROC-AUC associated with that value are reported. The results show that all architectures can achieve high detection performance, with F1-scores above 0.97 and ROC-AUC values close to 1.0 for objects 221 and 301 in most cases. This indicates a clear distinction between overshooting defects and non-defective parts. FoldingNet and PointNet++AE are consistent, attaining F1-scores around 0.98 for all three objects at very low contamination levels (between 0.001 and 0.003). This suggests their reconstruction error distributions allow Isolation Forest to operate with a conservative anomaly threshold. The VAE shows slightly more variability, especially for object 206. The optimal F1 score is 0.914, which is lower than the other objects. It is also reached at a higher contamination value of 0.062. This hints at a less distinct separation between normal and defective samples for this geometry. DGCNN shows the strongest contrast across objects. It achieves the highest F1 score (0.991) for object 301, but its performance drops for object 206 (0.795). Its optimal operating points for objects 206 and 221 require much larger contamination values (0.176 and 0.098, respectively). This behavior is consistent with the more irregular training curves observed earlier and suggests that while DGCNN can perform well on certain shapes, its anomaly scores are less stable across different sub-assemblies than those of FoldingNet and PointNet++AE.

**Figure 6:**
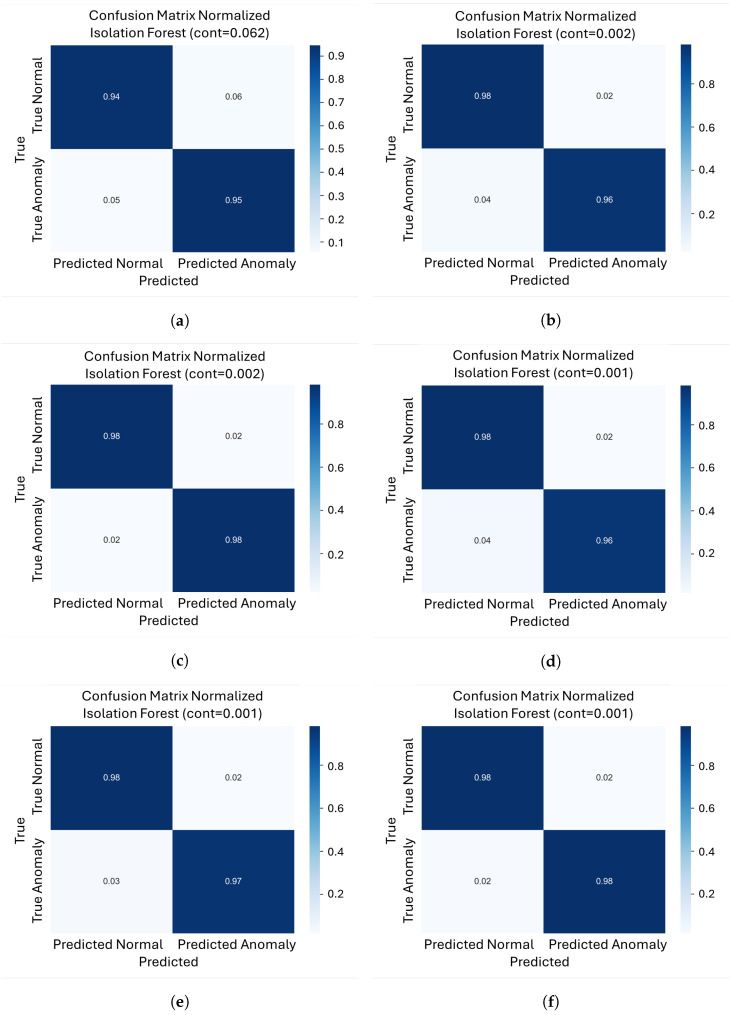

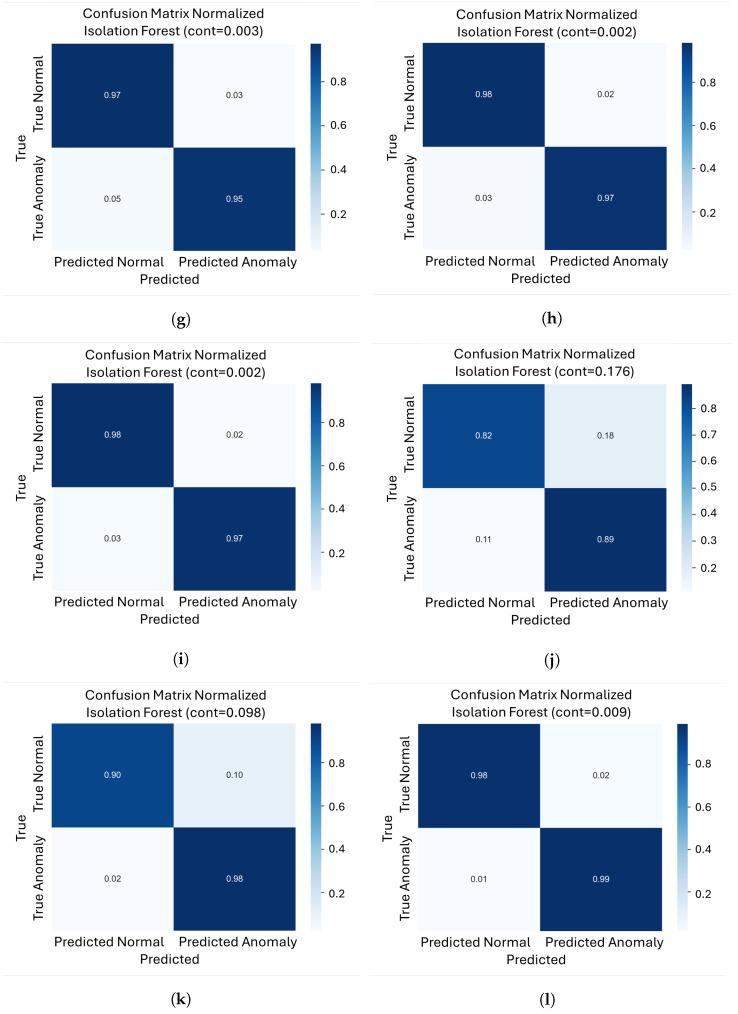
Normalized confusion matrices for our four architectures on the three different objects. (**a**) Normalized confusion matrix for object 206 with VAE; (**b**) Normalized confusion matrix for object 221 with VAE; (**c**) Normalized confusion matrix for object 301 with VAE; (**d**) Normalized confusion matrix for object 206 with FoldingNet; (**e**) Normalized confusion matrix for object 221 with FoldingNet; (**f**) Normalized confusion matrix for object 301 with FoldingNet; (**g**) Normalized confusion matrix for object 206 with PointNet++AE; (**h**) Normalized confusion matrix for object 221 with PointNet++AE; (**i**) Normalized confusion matrix for object 301 with PointNet++AE; (**j**) Normalized confusion matrix for object 206 with DGCNN; (**k**) Normalized confusion matrix for object 221 with DGCNN; (**l**) Normalized confusion matrix for object 301 with DGCNN.

To further evaluate how each model balances false positives and false negatives at its optimal operating point, we analyze the normalized confusion matrices (Figure 6a–l) for every model/object pair. These matrices were computed at the contamination level that maximizes the F1-score for each case.

For the VAE, object 301 shows very low error rates, with roughly 2% false positives and 2% false negatives. Object 221 exhibits similar behavior, with approximately 2% false positives and 4% false negatives. In contrast, object 206 exhibits higher misclassifications, with approximately 6% false positives and 5% false negatives, making it the most challenging case for this model.

For FoldingNet, all three objects exhibit consistently low error rates at very low contamination values. Objects 206, 221, and 301 show a balanced range of false positives (2–3%) and false negatives (3–4%), indicating an optimal compromise between missing defects and incorrectly flagging normal parts.

For PointNet++AE, Objects 301 and 221 had approximately 2% false positives and 3% false negatives. Object 206 has a slightly higher error rate, with around 3% false positives and about 5% false negatives.

For DGCNN, object 301 showed a very low false negative rate (around 1%) and a very low false positive rate (around 2%), indicating a strong tendency to detect almost all defects. However, for Object 221, the percentage of false positives increases to around 10%, while the percentage of false negatives remains at around 2%. The most extreme case is Object 206, which has approximately 18% false positives and 11% false negatives.

## 6. Conclusions and Future Works

Reconstruction-based models have become valuable tools for unsupervised anomaly detection, especially when labeled defective samples are limited. These architectures implicitly capture the typical geometric patterns of the underlying components by learning to encode and reconstruct only normal data. Anomalies are revealed as regions where the reconstruction differs from the input, providing a natural way to highlight defects without supervision. In the context of 3D point clouds, this approach is particularly appealing for quality control because it provides an automated method for identifying geometric deviations directly from the reconstructed shape. This study’s results showed that, when combined with Isolation Forest, reconstruction-based anomaly detection can achieve very high performance for overshooting defects. However, the results also revealed clear differences between architectures in terms of robustness and consistency.

Overall, FoldingNet and PointNet++ Autoencoder produced the most reliable results across the three objects, consistently achieving F1-scores close to 0.98 with very low contamination values. The VAE also delivered strong results for two of the objects but showed a more severe performance decline for the most challenging geometry (206). DGCNN exhibited the greatest variability; it produced the best result for object 301 but performed markedly worse for objects 206 and 221.

When examining each object individually, the behavior of the models becomes more specific. For object 301, DGCNN achieved the highest F1 score (0.991) and nearly perfect recall (0.998). This indicates that the dynamic graph representation is especially effective at separating normal and defective samples for this particular geometry. The other three models performed well on this object too, with F1-scores above 0.98. However, they did not match the combination of recall and overall F1 score achieved by DGCNN. For Object 221, FoldingNet and the PointNet++ Autoencoder performed similarly, both achieving F1 scores around 0.981 with high precision and recall. The VAE followed closely behind, while the DGCNN required a higher contamination level to reach an F1 score of 0.901. The differences are even more evident for object 206, where FoldingNet achieved the best result (an F1 score of 0.980) with an optimal balance of precision and recall. This was followed by the PointNet++ Autoencoder, which achieved an F1 score of 0.974. In contrast, the VAE dropped to 0.9145 and the DGCNN fell to 0.795, confirming the latter’s significant sensitivity to the subassembly’s specific geometry.

Taken together, these findings suggest that, although DGCNN can perform very well for specific shapes, such as Object 301, its behavior is less predictable across different components. Meanwhile, FoldingNet offers a more stable compromise. It consistently ranks among the top performers for all three objects, with high F1 scores, low required contamination, and relatively low model complexity. The PointNet++ Autoencoder also shows strong, consistent performance, particularly for objects 221 and 206, making it a solid alternative when a higher complexity model is acceptable.

From a practical point of view, our results indicate that FoldingNet is the most suitable default choice for overshooting detection in new shipbuilding sub-assemblies, as it offers a robust trade-off between accuracy, stability across geometries, and model complexity. PointNet++ Autoencoder can be recommended when a slightly higher computational cost is acceptable in exchange for comparable performance, particularly on geometries similar to objects 206 and 221, while DGCNN should be reserved for scenarios where its behavior can be validated on shapes close to object 301, given its strong dependence on the underlying geometry.

In conclusion, this study showed that reconstruction-based anomaly detection on 3D point clouds, combined with an unsupervised Isolation Forest classifier, is a viable strategy for identifying overshooting defects in shipbuilding sub-assemblies. Beyond the specific numerical results, the study shows the importance of choosing architectures that balance detection performance, stability across different geometries, and computational cost. These results can provide practical information for shipyards and subcontractors interested in integrating 3D reconstruction models into their quality control workflows.

For future work, we first suggest expanding the number and diversity of objects considered, including different types of sub-assemblies and defect geometries, to more thoroughly assess the generalization capability of the reconstruction models. We also suggest training and evaluating the full pipeline directly on point clouds acquired from real shipbuilding components instead of relying solely on 3D-printed representations. In addition, future work will focus on extending the proposed reconstruction-based approach from component-level defect detection to point-wise or region-wise defect localization on a 3D point cloud in order to better support defect localization and rework procedures in complex shipbuilding environments. Finally, we suggest integrating the best-performing models into an actual industrial inspection workflow to validate their performance under real-time constraints and evaluate their impact on shipyard quality control processes.

## Figures and Tables

**Table 1:** Performance of different models based on training time, parameters, and computational load.

	Average Training Time (s)	Trainable Parameters	Non-Trainable Parameters	Computational Load (GPU VRAM)
VAE	347.499	13,426,688	1408	45.9 GB
FoldingNet	437.27	602,755	0	46.5 GB
DGCNN	554.35	14,220,800	0	46.1 GB
PointNet++AE	935.29	28,403,776	11,264	46.2 GB

**Table 2:** Validation performance of the models for each object.

Model	Ref.	Contamination	F1-Score	Precision	Recall	ROC-AUC
	301	0.002	0.9871	0.996	0.9785	0.998
VAE	206	0.062	0.9145	0.884	0.946	0.977
	221	0.002	0.979	0.996	0.963	0.994
	301	0.001	0.983	0.998	0.965	0.995
FoldingNet	206	0.001	0.980	0.996	0.965	0.997
	221	0.001	0.981	0.997	0.965	0.997
	301	0.002	0.983	0.996	0.971	0.93
PointNet++ AE	206	0.003	0.974	0.994	0.954	0.996
	221	0.002	0.981	0.996	0.967	0.995
	301	0.009	0.991	0.982	0.998	0.994
DGCNN	206	0.176	0.795	0.7170	0.892	0.851
	221	0.098	0.901	0.837	0.0.994	0.931

## Data Availability

The original contributions presented in this study are included in the article material. Further inquiries can be directed to the corresponding author(s).

## References

[1] Carlsson B. (1989). The evolution of manufacturing technology and its impact on industrial structure: An international study. Small Bus. Econ..

[2] Lu D., Hui E.C.M., Shen J., Shi J. (2024). Digital industry agglomeration and urban innovation: Evidence from China. Econ. Anal. Policy.

[3] Zhao S., Zhang X. (2025). Did digitalization of manufacturing industry improved the carbon emission efficiency of exports: Evidence from China. Energy Strategy Rev..

[4] Marti L., Puertas R. (2023). Analysis of European competitiveness based on its innovative capacity and digitalization level. Technol. Soc..

[5] Bertagna S., Braidotti L., Bucci V., Marinò A. (2024). Laser Scanning Application for the Enhancement of Quality Assessment in Shipbuilding Industry. Procedia Comput. Sci..

[6] Wang H., Guo Y., Liang X., Yi H. (2019). A function-oriented quality control method for shipbuilding. Ships Offshore Struct..

[7] Yi Z., Mi S., Tong T., Li H., Lin Y., Wang W., Li J. (2023). Intelligent initial model and case design analysis of smart factory for shipyard in China. Eng. Appl. Artif. Intell..

[8] Salonen A., Gabrielsson M., Al-Obaidi Z. (2006). Systems sales as a competitive response to the Asian challenge: Case of a global ship power supplier. Ind. Mark. Manag..

[9] Mickeviciene R. (2011). Global competition in shipbuilding: Trends and challenges for Europe. The Economic Geography of Globalization.

[10] John L.S., Yoon S., Li J., Wang P. (2025). Anomaly Detection Using Convolutional Autoencoder with Residual Gated Recurrent Unit and Weak Supervision for Photovoltaic Thermal Heat Pump System. J. Build. Eng..

[11] Valero E., Forster A., Bosché F., Hyslop E., Wilson L., Turmel A. (2019). Automated defect detection and classification in ashlar masonry walls using machine learning. Autom. Constr..

[12] Ntoulmperis M., Catti P., Discepolo S., van de Kamp W., Castellini P., Nikolakis N., Alexopoulos K. (2024). 3D point cloud analysis for surface quality inspection: A steel parts use case. Procedia CIRP.

[13] Masuda M., Hachiuma R., Fujii R., Saito H., Sekikawa Y. (2021). Toward unsupervised 3d point cloud anomaly detection using variational autoencoder. Proceedings of the 2021 IEEE International Conference on Image Processing (ICIP).

[14] Du J., Tao C., Cao X., Tsung F. (2025). 3D vision-based anomaly detection in manufacturing: A survey. Front. Eng. Manag..

[15] Muzakir M., Ayob A., Irawan H., Pamungkas I., Pandria T., Fitriadi F., Hadi K., Amri A., Syarifuddin S. (2023). Defect analysis to improve quality of traditional shipbuilding processes in West Aceh District, Indonesia. AIP Conf. Proc..

[16] Ma H., Lee S. (2022). Smart system to detect painting defects in shipyards: Vision AI and a deep-learning approach. Appl. Sci..

[17] Hwang H.G., Kim B.S., Woo Y.T., Yoon Y.W., Shin S.C., Oh S.J. (2021). A Development of Welding Information Management and Defect Inspection Platform based on Artificial Intelligent for Shipbuilding and Maritime Industry. J. Korea Inst. Inf. Commun. Eng..

[18] Chen L., Yao X., Xu P., Moon S., Bi G. (2020). Rapid surface defect identification for additive manufacturing with in situ point cloud processing and machine learning. Virtual Phys. Prototyp..

[19] Zavrtanik V., Kristan M., Skočaj D. Cheating depth: Enhancing 3d surface anomaly detection via depth simulation. Proceedings of the IEEE/CVF Winter Conference on Applications of Computer Vision.

[20] Chen R., Xie G., Liu J., Wang J., Luo Z., Wang J., Zheng F. Easynet: An easy network for 3d industrial anomaly detection. Proceedings of the 31st ACM International Conference on Multimedia.

[21] Lhoste R., Vacavant A., Delhay D. (2025). MAESTRO: A Full Point Cloud Approach for 3D Anomaly Detection Based on Reconstruction. Proc. Copyr..

[22] Wang Z., Hotta K., Kamide K., Zou Y., Zhang C., Yu J. (2025). 3DKeyAD: High-Resolution 3D Point Cloud Anomaly Detection via Keypoint-Guided Point Clustering. arXiv.

[23] Kartashov O.O., Chernov A.V., Alexandrov A.A., Polyanichenko D.S., Ierusalimov V.S., Petrov S.A., Butakova M.A. (2022). Machine learning and 3D reconstruction of materials surface for nondestructive inspection. Sensors.

[24] Liu Y., Zhang C., Dong X., Ning J. (2025). Point cloud-based deep learning in industrial production: A survey. ACM Comput. Surv..

[25] Rani A., Ortiz-Arroyo D., Durdevic P. (2024). Advancements in point cloud-based 3D defect classification and segmentation for industrial systems: A comprehensive survey. Inf. Fusion.

[26] Intel RealSense Camera. https://www.intel.com/content/www/us/en/products/sku/205847/intel-realsense-depth-camera-d455/specifications.html.

[27] Yang Y., Feng C., Shen Y., Tian D. Foldingnet: Point cloud auto-encoder via deep grid deformation. Proceedings of the IEEE Conference on Computer Vision and Pattern Recognition.

[28] Qi C.R., Su H., Mo K., Guibas L.J. Pointnet: Deep learning on point sets for 3d classification and segmentation. Proceedings of the IEEE Conference on Computer Vision and Pattern Recognition.

[29] Wang Y., Sun Y., Liu Z., Sarma S.E., Bronstein M.M., Solomon J.M. (2019). Dynamic Graph CNN for Learning on Point Clouds. ACM Trans. Graph..

[30] Qi C.R., Yi L., Su H., Guibas L.J., Guyon I., Luxburg U.V., Bengio S., Wallach H., Fergus R., Vishwanathan S., Garnett R. (2017). PointNet++: Deep Hierarchical Feature Learning on Point Sets in a Metric Space. Proceedings of the Advances in Neural Information Processing Systems.

[31] Achlioptas P., Diamanti O., Mitliagkas I., Guibas L. Learning representations and generative models for 3d point clouds. Proceedings of the International Conference on Machine Learning, PMLR.

[32] Liu F.T., Ting K.M., Zhou Z.H. (2008). Isolation forest. Proceedings of the 2008 Eighth IEEE International Conference on Data Mining.

[33] Rubner Y., Tomasi C., Guibas L.J. (2000). The Earth Mover’s Distance as a Metric for Image Retrieval. Int. J. Comput. Vis..

[34] Bakshi A., Indyk P., Jayaram R., Silwal S., Waingarten E. (2023). A Near-Linear Time Algorithm for the Chamfer Distance. arXiv.

[35] Fan H., Su H., Guibas L.J. A point set generation network for 3d object reconstruction from a single image. Proceedings of the IEEE Conference on Computer Vision and Pattern Recognition.

